# Immune and body composition signatures of neurodevelopmental impairments in preterm preschoolers

**DOI:** 10.3389/fimmu.2026.1941190

**Published:** 2026-10-19

**Authors:** Armida Ivette Bahena-Delgado, Carmen Hernandez-Chavez, Sandra Martínez-Medina, Gabriela Gil-Martínez, Arturo Alejandro Canul-Euan, Ismael Mancilla-Herrera, Carolina Rodríguez-Hernández, Brenda Janeth Rojo-Prieto, Ameyali Mariana Rodríguez-Cano, Otilia Perichart-Perera, José Antonio Morales-González, Guadalupe Estrada-Gutierrez, Claudine Irles, Oscar Medina-Contreras, Gabriela Gonzalez-Perez

**Affiliations:** 1 Department of Physiology and Cellular Development, National Institute of Perinatology Isidro Espinosa de los Reyes, Mexico City, Mexico; 2 Instituto Politécnico Nacional, Escuela Superior de Medicina, Mexico City, Mexico; 3 Department of Developmental Neurobiology, National Institute of Perinatology Isidro Espinosa de los Reyes, Mexico City, Mexico; 4 Centro de Estudios Avanzados sobre Violencia-Prevención (CEAVI-P), Instituto Nacional de Pediatría, Mexico City, Mexico; 5 Star Médica Hospital Infantil Privado, Mexico City, Mexico; 6 Deputy Directorate of Biomedical Research, National Institute of Perinatology Isidro Espinosa de los Reyes, Mexico City, Mexico; 7 Coordination of Nutrition and Bioprogramming, National Institute of Perinatology Isidro Espinosa de los Reyes, Mexico City, Mexico; 8 Department of Immunobiochemistry, National Institute of Perinatology “Isidro Espinosa de los Reyes”, Mexico City, Mexico; 9 INSERM UMR 1349, Bobigny, France; 10 Laboratoire Signalisation Microenvironnement et Hémopathies Lymphoïdes B (SIMHEL), Université Sorbonne Paris Nord, Bobigny, France; 11 Immunometabolism Laboratory, Hospital Infantil de Mexico Federico Gomez, Mexico City, Mexico

**Keywords:** neurodevelopment, neutrophils, platelets, preschoolers, preterm, subcutaneous adiposity, transitional B cells

## Abstract

**Background:**

Preterm birth is a primary risk factor for long-term neurodevelopmental deficits. While acute neonatal inflammation is a known disruptor of brain development, the persistence of immune dysregulation and its relationship with body composition in the preschool years remains poorly characterized. This study aimed to identify immunological and anthropometric signatures associated with neurodevelopmental outcomes in preschoolers born preterm.

**Methods:**

We conducted a prospective cohort study of preschoolers born at ≤ 34 weeks gestation. We performed comprehensive anthropometry, high-dimensional flow cytometry, and quantification of serum neuroinflammatory biomarkers, on children with Normal, Delay or Risk in neurodevelopment.

**Results:**

Neurodevelopmental impairments predominantly affected motor, language and cognitive domains in our cohort. Children in the Delay and Risk groups exhibited significantly reduced subcutaneous adiposity and lower fat mass compared to the Normal group, despite comparable linear growth. Immunologically, the Risk group was characterized by decreased neutrophils, platelets, and IP-10 levels. The B-cell compartment was the strongest predictor of clinical status; children with neurodevelopmental deficits exhibited a persistence of immature transitional B cells (CD38^+^CD24^+^IgD^+^), whereas those with normal development were enriched for mature class-switched memory B cells (CD27^+^IgD^-^).

**Conclusion:**

Preterm preschoolers with neurodevelopmental impairments showed a systemic profile of biological immaturity (reduced subcutaneous adiposity, lower neutrophil and platelet counts and a persistence of transitional B cells). Our identification of a distinct B-cell signature and altered body composition profiles in preschoolers born preterm offers promising new avenues for early detection and intervention.

## 1 Introduction

Preterm birth remains a significant global health issue that accounts for approximately 16% of all births, totaling 13.4 million worldwide (1–4). Preterm neonates are a vulnerable population exposed to adverse intrauterine and postnatal events that interrupt organ development during critical growth stages (5). Consequently, preterm survivors face increased risk of long-term comorbidities, including immunological disorders, delayed growth, altered body composition, and neurodevelopmental impairments (3, 4, 6).

A significant challenge in these children is encephalopathy of prematurity, a condition characterized by brain injury, often triggered by hypoxia-ischemia, inflammation, and infection during fetal and postnatal stages, that can result in neurodevelopmental disorders later in life (7). While the central nervous system (CNS) is traditionally considered an immunologically privileged site, it can be severely affected by systemic infections occurring during pregnancy or the postnatal period (8). Furthermore, the immaturity of the preterm brain renders their CNS particularly susceptible to the hostile environment of the Neonatal Intensive Care Unit (NICU), where persistent stressors and inflammatory events can lead to permanent neurodevelopmental alterations (9–12).

Tissue dysfunction, rather than just acute damage, can trigger persistent inflammatory processes, and even after the primary stimuli ceases, a cycle of inflammation-dysfunction may establish low-grade chronic inflammation with long-term systemic repercussions (13). Within this inflammatory context, immune cells such as T cells, B cells, and antigen-presenting cells are of particular interest; while they do not normally reside in the brain, they may modulate neural function by crossing the blood-brain barrier (BBB) or releasing soluble factors, particularly in preterm infants with altered BBB permeability (14–17).

Neurodevelopmental disorders may clearly manifest in children between 3 and 5 years, as this is a sensitive period for the consolidation of cognitive, motor, and language functions (18). Prematurity has been linked to a significantly higher risk of motor, language, cognitive, and psychiatric disorders. However, most preterm studies focus on children under three years, school-age children, and adolescents (19, 20), despite evidence showing that functional difficulties frequently manifest at preschool age (21–23). Biological immaturity and anthropometric alterations associated with prematurity can disturb the mechanisms of postnatal neurobiological development, resulting in poor performance during neurodevelopmental assessments, even in the absence of early neurological complications (24).

The assessment of neuroimmune inflammation biomarkers (25), together with the evaluation of growth rates and body composition (26, 27), hold promise for early prediction of adverse neurodevelopmental outcomes in preterm infants (28).

The aim of this study was to characterize the body composition and immunological profile of preschool children born preterm and their association with neurodevelopmental outcomes.

## 2 Materials and methods

### 2.1 Research design and study population

Observational prospective longitudinal study with a cohort design with eligibility criteria of children born preterm at the National Institute of Perinatology “Isidro Espinosa de los Reyes” (INPer) between March 2018 and May 2020. NEOPREM (ImmuNe system-intEstinal micrObiota-brain axis and neurodeveloPment of infants born pREterM from birth to age five) cohort recruited children 42 to 48 months old between November 2021 and November 2023 and followed up until 60 months. Inclusion criteria were female and male children, born at ≤ 34 weeks gestational age by vaginal birth and cesarean section from mothers > 18 years. Exclusion criteria were children with major congenital defects, autoimmune and genetic diseases, cerebral palsy, non-residents of Mexico City and Metropolitan Area. Elimination criteria were children diagnosed with intellectual disability during neurodevelopmental assessments, and children without blood samples. For the analysis, children were classified in 3 study groups according to their results in the Child Development Evaluation Test Second Edition (EDI-II): Normal (n = 17), Delay (n = 27), and Risk (n = 21).

### 2.2 Data collection

Infant and maternal data were retrieved from electronic medical records. Infant variables included: mode of delivery (programmed C-section, emergency C-section, eutocic birth, dystocic birth), gestational age (GA), sex, parity (singleton, twins, triplets), birth weight, Apgar at 1 and 5 minutes, invasive mechanical ventilation (IMV), length of hospitalization, antibiotic (ABT) administration during hospitalization. Infant clinical diagnoses during hospitalization included: early-onset sepsis (EOS), late-onset sepsis (LOS), necrotizing enterocolitis (NEC) II and III, atypical pneumonia, moderate and severe bronchopulmonary dysplasia (BDP), apnea, retinopathy or prematurity (ROP), multifactorial hyperbilirubinemia (MFHB), brain hemorrhage (intraventricular, intracerebral, subependymal, intraparenchymal, periventricular). Maternal variables included maternal age, years of education, marital status (single, married, consensual union), socioeconomic status (low, medium), pregestational nutritional status according to body mass index (BMI) (healthy weight, overweight, obesity), preeclampsia.

### 2.3 Neurodevelopmental assessment

Neurodevelopmental assessments were conducted in distraction-free environments by certified psychologists from the Department of Developmental Neurobiology at INPer that were blinded to the patients’ medical history (see **Supplementary Methods** for details).

Screening assessment: All preschoolers (n = 65) were evaluated with the Child Development Evaluation Test Second Edition (EDI-II, by its acronym in Spanish) between 42- and 48-months, and the average assessment duration was 15 minutes. The EDI-II test was designed and validated for Mexican children aged 1–60 months, assesses five domains (gross motor, fine motor, language, social, and cognitive skills), explores the general neurological status, identifies biological risk factors related to prenatal care and neonatal complications, and addresses warning signs. Results are classified by a traffic-light system: Green (Normal development), Yellow (Developmental Lag, hereafter referred as Delay), and Red (Risk of developmental delay, hereafter referred as Risk) (29, 30).

Diagnostic Assessment: Children’s neurodevelopment was assessed between 42- and 48-months (n = 61), and at 60 months (n = 55) using the McCarthy Scales of Children’s Abilities (MSCA), and the average assessment duration was 45 to 60 minutes. The MSCA test evaluates six domains in children aged 2.5-8.5 years: verbal, perceptual-performance, quantitative, memory, motor, and general cognitive index (GCI, a composite score derived from the verbal, perceptual-performance and quantitative scales). Domain scores have a mean of 50 ± 10, except for the GCI, which has a mean of 100 ± 15. Raw values were standardized by child age. Higher scores indicate better developmental outcomes (**Supplementary Figure 8**) (22, 31–33).

### 2.4 Nutritional and anthropometric assessment

Anthropometric measures were performed in duplicate by experienced and trained nutritionists following Lohman’s methodology (34), as previously described for infants (35). Measurements included weight (Digital scale SECA 876), height (Stadiometer SECA 264), and circumferences (head, arm, abdomen, thigh, calf) using a W606PM Lufkin tape (Apex Tool Group). Skinfold thicknesses (biceps, triceps, subscapular, abdominal, thigh, calf) were measured using a Lange caliper (Beta Technology).

Calculated Indices: Body Mass Index (BMI): Weight (kg)/Height (m^2^); Waist-to-Height Ratio (WHR): Abdominal Circumference (m)/Height (m) (36, 37); Arm Muscle Area (AMA): [MUAC - (π x TSF)]^2^/(4 x π)], where MUAC is the mid-upper arm circumference and TSF is triceps skinfold thickness; Central-to-Total Skinfold Ratio: SSF+ASF/ΣSF, where SSF is subscapular and ASF is abdominal skinfold thickness, and ΣSF is the sum of biceps, triceps, subscapular, abdominal, thigh, calf skinfolds thickness.

Body Composition: Percentage of Fat Mass (% FM) was estimated using Slaughter’s equations for Girls: 1.33 x (TSF+SSF) - 0.013 x (TSF+SSF)^2^ - 2.5 and Boys: 1.21 x (TSF+SSF) - 0.008 x (TSF+SSF)^2^ - 1.7 (38); Absolute Fat Mass (kg): [(% FM/100) x Weight (kg)]; Fat-Free Mass (FFM): Weight (kg) - FM (kg).

Dietary Intake: A food frequency questionnaire was administered to the primary caregiver to assess daily meal frequency and consumption of specific food groups and beverages. Standardized measuring cups and spoons were used to estimate portions.

### 2.5 Blood sampling, hematic biometry and blood chemistry

Peripheral blood was collected in K_2_EDTA (500 µL) and SST Amber tubes (600 µL) (BD Microtainer) at 42–48 months old when EDI-II test was administered. K_2_EDTA blood samples were used for hematic biometry (Beckman Coulter DxH 500) and immunophenotyping by flow cytometry. Serum samples were used for blood chemistry (glucose, triglycerides, total cholesterol, HDL-cholesterol, creatinine, uric acid and urea) with a dry chemistry analyzer (Respons^®^910 DiaSys), and quantification of neuroimmune inflammation biomarkers by Multiplex Immunoassays.

### 2.6 Immunophenotyping

Innate (NK cells) and adaptive (T cells and B cells) immune populations were analyzed using 70 µL of peripheral blood per panel. Samples were washed with PBS, stained with Zombie Aqua fixable viability dye (BioLegend), blocked with human TrueStain FcX (BioLegend), incubated with fluorochrome-conjugated monoclonal antibodies against CD14 (M5E2), CD16 (3G8), CD56 (HCD56), CD8α (RPA-T8), CCR7 (G043H7), CD45RA (HI100), CD4 (RPA-T4), CD3 (UCHT1), IgD (IA6-2), CD24 (ML5), CD20 (2H7), CD27 (M-T271), CD38 (HIT2), CD19 (HIB19), HLA-DR (L243) for 30 min at room temperature (all antibodies from BioLegend), and red blood cells were lysed with BD FACS Lysing Solution (BD Biosciences). Data were acquired on a BD FACSAria III flow cytometer and analyzed using FlowJo v10. Gating strategies are detailed in **Supplementary Figures 5**–**7**.

### 2.7 Computational flow cytometry analysis

Briefly, prior to clustering, raw .fcs files were evaluated using FlowJo software to exclude debris, doublets, and dead cells via a Fixable Viability Dye (FVD). Only single, viable, intact mononuclear cells were exported as cleaned .fcs files and loaded into the CATALYST environment for subsequent high-dimensional analysis.

Preprocessing: Raw .fcs files were processed in R (v4.5.2) using flowCore and CATALYST. Channel names were standardized, and fluorescence intensities were arcsinh-transformed (cofactor 150).

Dimensionality Reduction & Clustering: UMAP was performed on downsampled data (1,000 cells/sample) for visualization. Unsupervised clustering was conducted using FlowSOM (10×10 grid) followed by ConsensusClusterPlus metaclustering to define 15 metaclusters per panel.

Annotation & Differential Abundance: Metaclusters were auto-annotated based on median marker expression thresholds (≥ 2.0 considered positive). Differential abundance between clinical groups (Normal vs. Risk/Delay) was assessed using generalized linear models (GLM) via edgeR with Benjamini-Hochberg FDR correction.

Correlation & Predictive Modeling (RAPID): Spearman rank correlation identified cell populations tracking with clinical severity (Normal=1, Delay=2, Risk=3). To identify a minimal immune signature predictive of clinical outcome, we applied a machine learning approach using LASSO (Least Absolute Shrinkage and Selection Operator) regularized logistic regression, implemented via the glmnet package in R (39). The LASSO model performs feature selection by applying a penalty that shrinks the regression coefficients of non-informative features to exactly zero, thereby selecting only the most robust predictors and preventing overfitting. The optimal regularization parameter (λ) was determined through k-fold cross-validation (with k adapted to sample size), which minimizes the model’s prediction error (Binomial Deviance). Clusters with non-zero coefficients at this optimal λ were identified as the final predictive immune signature. To evaluate the performance of the final model, we calculated standard classification metrics on the training set itself, as sample size limitations precluded the use of an independent hold-out test set. The model’s predictions on the training data were used to compute Accuracy, Sensitivity (the proportion of true Delay/Risk cases correctly identified), and Specificity (the proportion of true Normal cases correctly identified) (see **Supplementary Methods** for details).

### 2.8 Neuroimmune inflammation biomarkers assessment

Serum biomarkers were quantified using two bead-based immunoassays (LEGENDplex, BioLegend) according to the manufacturer’s instructions: 1) Essential Immune Response Panel: IL-1β, IL-2, IL-4, IL-8, IL-10, IL-12p70, IL-17A, IFN-γ, IP-10 (n=60); 2) Neuroinflammation Panel: BDNF, CCL2 (MCP-1), CX3CL1, IL-6, IL-18, sRAGE, sTREM-1, sTREM2, TNF-α, TGF-β1, VILIP-1, VEGF, β-NGF (n=57). Sample sizes varied due to serum availability. Data was acquired on a FACSAria III flow cytometer and analyzed using QOGNIT software.

### 2.9 Statistical analysis

Statistical analysis of demographic, clinical, nutritional, environmental, and maternal variables from studied infants were performed using GraphPad Prism version 10.4.1. Descriptive statistics are reported as mean ± standard deviation (SD) for normal continuous variables, median [25th percentile-75th percentile] for non-normal continuous variables, or frequencies (%) for categorical variables. Normality was assessed using the Shapiro–Wilk test. Group comparisons were performed using one-way ANOVA with Šídák’s *post hoc* test for normally distributed variables or Kruskal–Wallis test with Dunn’s *post hoc* test for non-normally distributed variables. Fisher’s exact test and chi-squared test were used to compare the frequencies of categorical variables as appropriate. A *p*-value < 0.05 was considered statistically significant. Correlations between MSCA scores, anthropometry, and biomarkers were evaluated using Spearman’s rank correlation.

## 3 Results

### 3.1 Preschoolers born preterm exhibit persistent impairments in motor, language, and cognitive domains

Sixty-five preschoolers born preterm from the NEOPREM cohort were included in the analysis, and their neurodevelopment was evaluated with a screening test (Child Development Evaluation Test Second Edition, EDI-II), and a diagnostic test (McCarthy Scales of Children’s Abilities, MSCA) between 42 to 60 months (**Figure 1**).

**Figure 1:**
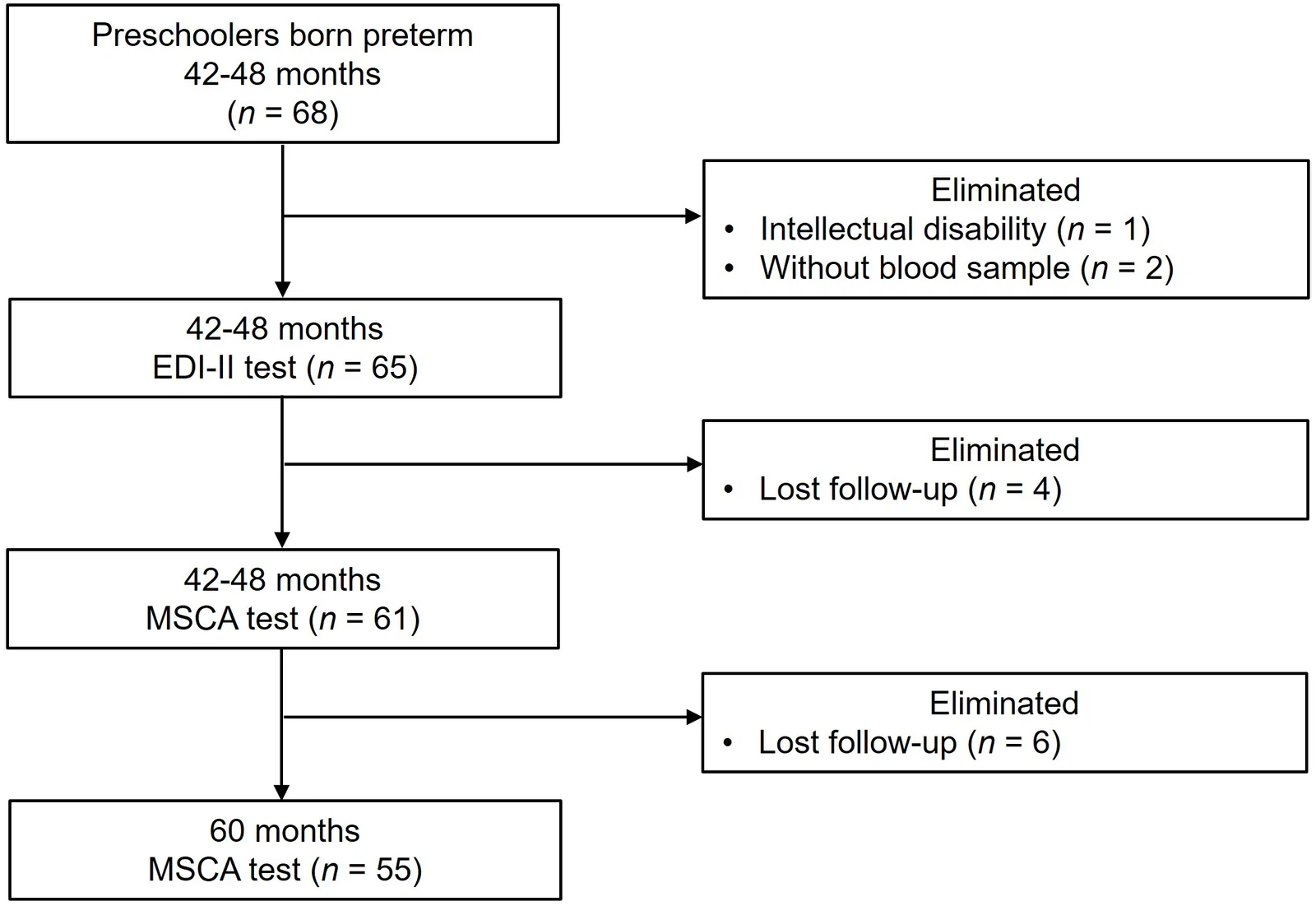
Flow diagram of preschoolers from the NEOPREM cohort included in the study. Preschoolers were evaluated with the Child Development Evaluation Test Second Edition (EDI-II) between 42- and 48-months (*n* = 65), and with the McCarthy Scales of Children’s Abilities (MSCA) between 42- and 48-months (*n* = 61) and at 60 months (*n* = 55).

The global result of EDI-II test was used to evaluate the overall performance of children at 42–48 months in five neurodevelopmental domains (gross motor, fine motor, language, social, and cognitive), and to classify them in three groups: Normal (n = 17, 26.2%), Delay (n = 27, 41.5%), and Risk (n = 21, 32.3%) (**Figure 2A**). Within the Normal group, 76.5% were females, whereas in the Delay and Risk groups the proportion of females and males was similar (female 55.6% vs male 44.4% in the Delay group; female 42.9% vs male 57.1 in the Risk group); no statistical differences between groups were found (*p* = 0.113) (**Table 1**). EDI-II results analysis by neurodevelopmental domain revealed that children presented Delay/Risk performance in gross motor (46.1%), cognitive (33.9%), language (33.8%), fine motor (26.1%) and social skills (21.5%) (**Figure 2A**, **Supplementary Table 1**).

**Figure 2:**
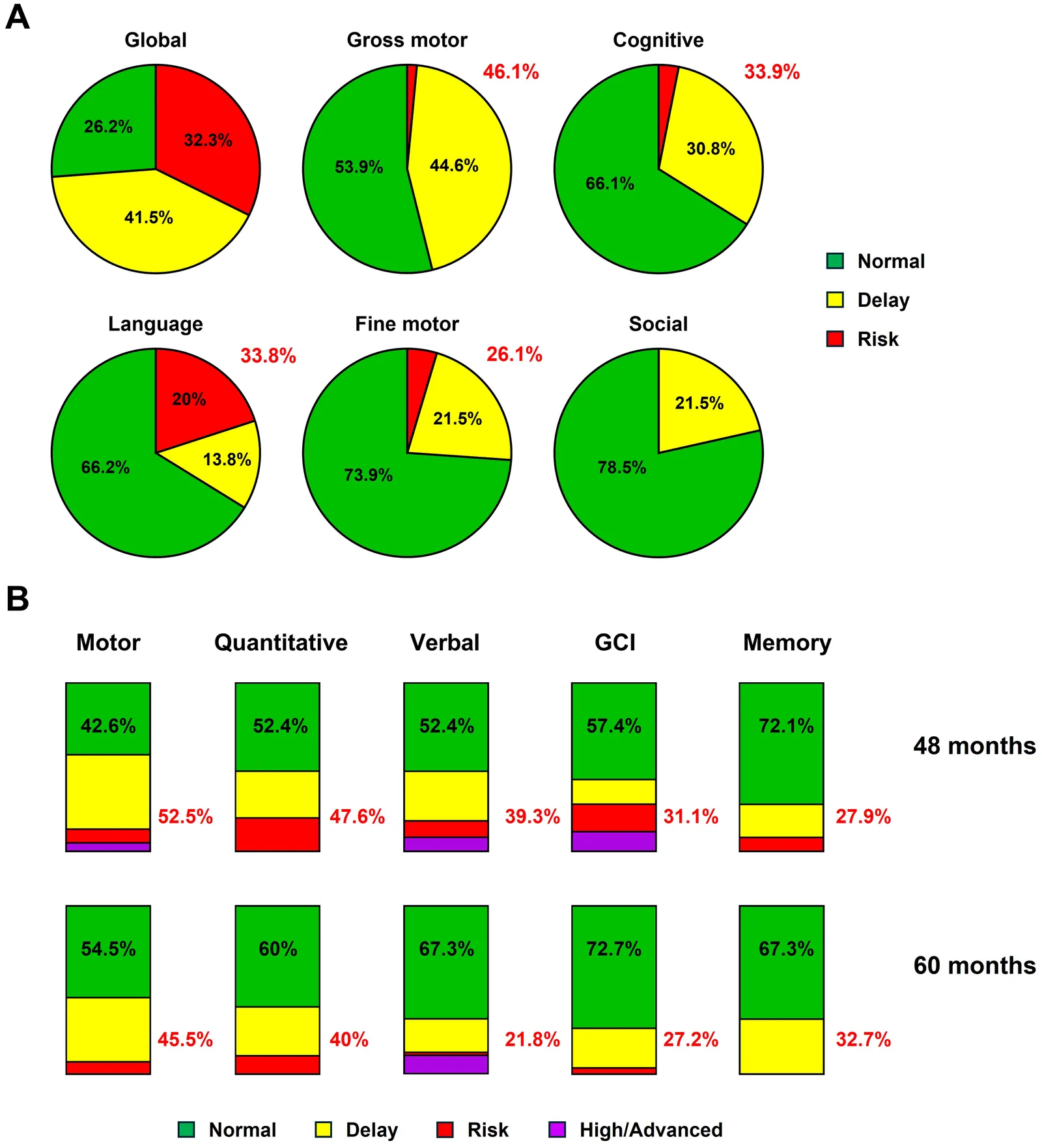
Neurodevelopmental assessment of preschoolers born preterm. **(A)** Results from the EDI-II test at 42–48 months; the global result (Normal *n* = 17, 26.2%, green; Delay *n* = 27, 41.5%, yellow; Risk *n* = 21, 32.3%, red) as well as the results per domain (gross motor, cognitive, language, fine motor, and social) are shown. The red value next to the pie charts represents the sum of percentages of the Delay and Risk groups. **(B)** Results from the MSCA test (motor, quantitative, verbal, general cognitive index, and memory domains are shown) at 48 months (top) and 60 months (bottom). Normal (green), delay (yellow), risk (red), and the sum of high and advanced (lilac) results are shown as percentages. The red value next to the stacked bars represents the sum of percentages of the delay and risk groups.

**Table 1:** Demographic, clinical, and maternal data from studied preschoolers.

Neonatal variable	Normal (*n* = 17)	Delay (*n* = 27)	Risk (*n* = 21)	*p*-value
GA (weeks)	32.3 [29-33]	30.3 [28-32.3]	32 [28.3-33.5]	0.232
Female (%)	13 (76.5)	15 (55.6)	9 (42.9)	0.113
Male (%)	4 (23.5)	12 (44.4)	12 (57.1)	0.113
Birth weight (g)	1325 ± 294.4	1288 ± 494.7	1305 ± 422.6	0.961
IMV (%)	3 (17.6)	11 (40.7)	6 (28.5)	0.261
Antibiotic days	3 [0-15.5]	5 [0-22]	7 [0-22]	0.392
Hospitalization days	46.5 ± 20.1	61.9 ± 33.9	51.6 ± 32.4	0.235
EOS (%)	5 (29.4)	13 (48.1)	9 (42.8)	0.465
LOS (%)	4 (23.5)	8 (29.6)	6 (28.6)	0.902
Atypical pneumonia (%)	2 (11.8)	9 (33.3)	6 (28.6)	0.271
NEC (%)	3 (17.6)	2 (7.4)	4 (19)	0.434
BPD (%)	6 (35.3)	11 (40.7)	8 (38.1)	0.936
Apnea (%)	8 (47)	9 (33.3)	8 (38.1)	0.660
MFHB (%)	14 (82.3)	25 (92.6)	17 (80.9)	0.434
Brain hemorrhage (%)	4 (23.5)	5 (18.5)	6 (28.6)	0.713
ROP (%)	3 (17.6)	10 (37)	5 (23.8)	0.334
Maternal variable				
Maternal age (years)	32 [26-35]	28 [24-34]	26 [22-35.5]	0.534
PG healthy weight (%)	9 (52.9)	12 (44.4)	10 (47.6)	0.859
PG overweight (%)	6 (35.3)	11 (40.7)	6 (28.6)	0.682
PG obesity (%)	2 (11.8)	4 (14.8)	5 (23.8)	0.643
Preeclampsia (%)	9 (52.9)	5 (18.5)	10 (47.6)	**0.033**

GA, gestational age; IMV, invasive mechanical ventilation; EOS, early-onset sepsis; LOS, late-onset sepsis; BPD, bronchopulmonary dysplasia; MFHB, multifactorial hyperbilirubinemia; PG, pregestational; ROP, retinopathy of prematurity. Data are presented as mean ± standard deviation or median [interquartile range]. One-way ANOVA or Kruskal-Wallis tests were used for continuous variables. Fisher's exact test or Chi-Square test were used for categorical variables. Statistically significant *p*-values (*p* < 0.05) are shown in bold.

To confirm the EDI-II test results and to perform a deep analysis of children’s neurodevelopmental skills and follow up at 60 months, the MSCA test was administered. At 42–48 months old, the most affected domains with Delay/Risk scores were motor (52.5%), quantitative (47.6%), verbal (39.3%), general cognitive index (31.1%), and memory (27.9%) domains (**Figure 2B**, **Supplementary Table 2**). Subsequent evaluation at 60 months old revealed a persistent motor Delay/Risk (45.5%), followed by quantitative impairments (40%) and memory delay (32.7%) (**Figure 2B**, **Supplementary Table 3**).

### 3.2 Neonatal clinical history and maternal sociodemographic profiles

Most preschoolers were delivered by emergency C-section with a median gestational age between 30 to 32 weeks, and we did not observe differences in sex, birth plurality (singleton, twins, triplets), Apgar at 1 and 5 minutes, birth weight, antibiotic days administered during hospitalization, IMV use, and hospitalization length among preschoolers (**Table 1**, **Supplementary Table 4**). Next, we analyzed if early-life clinical diagnoses related with encephalopathy of prematurity (infection, inflammation, brain injury) were associated with EDI-II results, and found similar frequencies of EOS, LOS, atypical pneumonia, NEC, BPD, apnea, MFHB, brain hemorrhage and ROP among the 3 study groups (**Table 1**).

We did not observe differences in maternal age and pregestational nutritional status, and whereas about 50% of mothers in the Normal and Risk groups had preeclampsia during pregnancy there was 18.5% within the Delay group (**Table 1**).

Most preschoolers belonged to families with low socioeconomic status (SES), but about 30% of children in the Delay group had a medium SES. All mothers had a median schooling length of 12 years, and we did not observe differences in marital status (**Supplementary Table 4**).

### 3.3 Neurodevelopmental delay and risk are associated with reduced subcutaneous adiposity despite a healthy nutritional status

To assess the nutritional status of preschoolers we performed a food consumption frequency assessment, hematic biometry, blood chemistry, anthropometric and body composition measures.

We did not observe differences in feeding patterns (**Supplementary Table 5**); white and red blood cell counts, % hematocrit and hemoglobin concentration (**Table 2**); blood chemistry analytes (**Supplementary Table 6**); weight, height, BMI, WHR and AMA among children (**Table 3**). These results suggested that preschoolers had a healthy nutritional status overall despite the observed neurodevelopmental impairments.

**Table 2:** Hematic biometry from studied preschoolers.

Parameters	Normal (*n* = 17)	Delay (*n* = 27)	Risk (*n* = 21)	*p*-value
WBC (10^3^/μL)	7.5 ± 2	6.1 ± 1.6	6.5 ± 2.4	0.097
RCB (10^3^/μL)	4.9 ± 0.3	4.8 ± 0.3	4.8 ± 0.3	0.320
Hemoglobin (g/dL)	13.7 ± 0.8	13.7 ± 0.9	13.6 ± 0.7	0.917
Hematocrit (%)	41.2 ± 2.5	40.2 ± 2.4	40.2 ± 2.4	0.361
Neutrophils (10^3^/μL)	2.7 [2-3.7]	1.8 [1.3-2.6]	1.7 [0.9-2.7]*	**0.035**
Lymphocytes (10^3^/μL)	4 ± 1.3	3.5 ± 0.9	3.9 ± 1.2	0.342
Monocytes (10^3^/μL)	0.4 [0.2-0.6]	0.3 [0.3-0.5]	0.3 [0.2-0.4]	0.680
Eosinophils (10^3^/μL)	0.1 [0.0-0.3]	0.1 [0.0-0.1]	0.0 [0.0-0.2]	0.215
Basophils (10^3^/μL)	0.06 [0.03-0.07]	0.04 [0.03-0.07]	0.06 [0.04-0.08]	0.209
Platelets (10^3^/μL)	340 [293.5-400.5]	286 [220-323]*	266 [218.5-337.5]*	**0.021**
NLR	0.7 ± 0.5	0.6 ± 0.2	0.5 ± 0.4*	**0.048**
PLR	98.7 ± 42.3	82.6 ± 33.1	73.5 ± 33.2	0.102

WBC, White Blood Cell Count; RBC, Red Blood Cell Count; NLR, Neutrophil-to-Lymphocyte Ratio; PLR, Platelet-to-Lymphocyte Ratio. Data are presented as mean ± standard deviation or median [interquartile range]. One-way ANOVA or Kruskal-Wallis tests were used for continuous variables. Fisher's exact test or Chi-Square test were used for categorical variables. Statistically significant *p*-values (*p < 0.05) are shown in bold.

**Table 3:** Anthropometric and body composition data from studied preschoolers.

Parameters	Normal (*n* = 16)	Delay (*n* = 24)	Risk (*n* = 20)	*p*-value
Weight (kg)	14.3 ± 2.7	14.0 ± 2.1	13.2 ± 1.6	0.311
Height (cm)	98.4 [93-101.3]	97.3 [94.4-99.5]	96.6 [92.3-98.2]	0.449
BMI (kg/m^2^)	14.9 [13.6-16]	14.8 [14.3-15.5]	14.4 [13.8-15.2]	0.469
WHR	0.48 ± 0.02	0.48 ± 0.02	0.47 ± 0.02	0.129
AMA (cm^2^)	12.9 ± 2.1	13.8 ± 2.2	13.1 ± 2.1	0.374
∑SF (mm)	54.8 ± 15.6	45.3 ± 9.8*	43 ± 7.4*	**0.009**
SSF+ASF/∑SF	0.27 ± 0.03	0.25 ± 0.03	0.24 ± 0.03	0.056
FM (%)	16.5 ± 5.1	13.3 ± 3.6*	12.2 ± 2.6*	**0.005**
FM (kg)	2.4 ± 1.1	1.9 ± 0.6	1.6 ± 0.4*	**0.008**
FFM (kg)	11.8 ± 1.8	12.4 ± 1.6	11.5 ± 1.4	0.526
Circumference	Normal (*n* = 17)	Delay (*n* = 25)	Risk (*n* = 21)	*p*-value
Head (cm)	48.8 ± 1.8	48.8 ± 1.5	47.8 ± 1.6	0.115
Arm (cm)	15.2 ± 1.5	15.2 ± 1.3	14.8 ± 1.1	0.501
Abdominal (cm)	47 ± 3.2	46.6 ± 2.7	44.7 ± 1.9*	**0.027**
Thigh (cm)	27.5 [24.8-29.4]	27.4 [25-28.4]	27.1 [25-28.6]	0.784
Calf (cm)	20.1 [17.6-22.7]	19.7 [18.1-20.9]	19.5 [18.3-20.1]	0.564
Skinfold	Normal (*n* = 16)	Delay (*n* = 24)	Risk (*n* = 20)	*p*-value
Biceps (mm)	6 [4.5-6.3]	5 [4-5.5]	4.5 [4-5]	0.070
Triceps (mm)	8.1 ± 2.4	6.8 ± 1.8	6.5 ± 1.3*	**0.035**
Subscapular (mm)	6 [5-7.5]	5 [4.5-6]	5 [4.1-5.5]*	**0.031**
Abdominal (mm)	8.6 ± 2.9	6.3 ± 2.1*	5.7 ± 1.1*	**0.0006**
Thigh (mm)	13.7 [11.1-16.5]	12.7 [10.1-14.8]	11.2 [8.6-14.3]	0.232
Calf (mm)	10 [8-12.5]	9.5 [8.5-10.7]	9.2 [8-11]	0.722

BMI, Body Mass Index; WHR, Waist-to-height ratio; AMA, Arm muscle area; SSF, Subscapular Skinfold; ASF, Abdominal Skinfold; ∑SF, Total Skinfold; FM, Fat mass; FFM, Fat free mass. Data are presented as mean ± standard deviation or median [interquartile range]. One-way ANOVA or Kruskal-Wallis tests were used for continuous variables. Statistically significant *p*-values (*p < 0.05) are shown in bold.

Remarkably, body composition assessment revealed that children in the Delay and Risk groups had a reduced sum of skinfolds thickness (subcutaneous adiposity), % fat mass, and abdominal skinfolds thickness compared to children in the Normal group (**Table 3**). However, the waist-to-height ratio (WHR) showed no significant difference among groups (*p* = 0.129). This indicates that while children in the Risk group had lower triceps and subscapular skinfolds, and abdominal circumference than those in the Normal group (**Table 3**), the lack of difference in WHR suggests a nuanced compartmentalization of adipose tissue—a leaner phenotype—rather than a uniform reduction across all central adiposity indices.

### 3.4 Neurodevelopmental delay and risk are associated with altered myeloid profiles and reduced neutrophil-to-lymphocyte ratio

Detailed analysis of peripheral blood cell counts revealed that preschoolers from the Delay and Risk groups exhibited lower platelet counts than children in the Normal group (**Table 2**). Furthermore, children in the Risk group had reduced neutrophil counts, and lower neutrophil-to-lymphocyte ratios (NLR) than those with Normal and Delayed development (**Table 2**). Lymphocytes, monocytes, eosinophils, basophils, and platelet-to-lymphocyte ratios (PLR) were similar among children (**Table 2**). Despite the observed differences, peripheral blood cell counts remained within normal physiological values.

### 3.5 B-cell maturation is delayed in children with neurodevelopmental deficits, while T and NK compartments remain unchanged

To further assess the status of innate and adaptive immune cells compartments in the preschoolers, we immunophenotyped peripheral blood NK cells, T cells, and B cells by flow cytometry.

All preschoolers had a similar frequency of NK cell subpopulations (CD56^dim^CD16^+^, CD56^hi^CD16^-^, CD56^-^CD16^+^) (**Supplementary Figure 1**), as well as CD4^+^ and CD8^+^ T cell subpopulations (CD45RA^+^CCR7^+^ naïve, CD45RA^-^CCR7^+^ central memory, CD45RA^-^CCR7^-^ effector memory, and CD45RA^+^CCR7^-^ EMRA) (**Supplementary Figure 2**).

Interestingly, within the B-cell compartment, children in the Delay group exhibited higher frequency of transitional B cells (CD38^hi^CD24^+^) than those in the Normal group, whereas the frequencies of plasmablasts (CD38^+^CD24^-^), naive (IgD^+^CD27^-^), non-class-switched memory (IgD^+^CD27^+^), and class-switched memory (IgD^-^CD27^+^) B cells were similar among groups (**Figure 3**).

**Figure 3:**
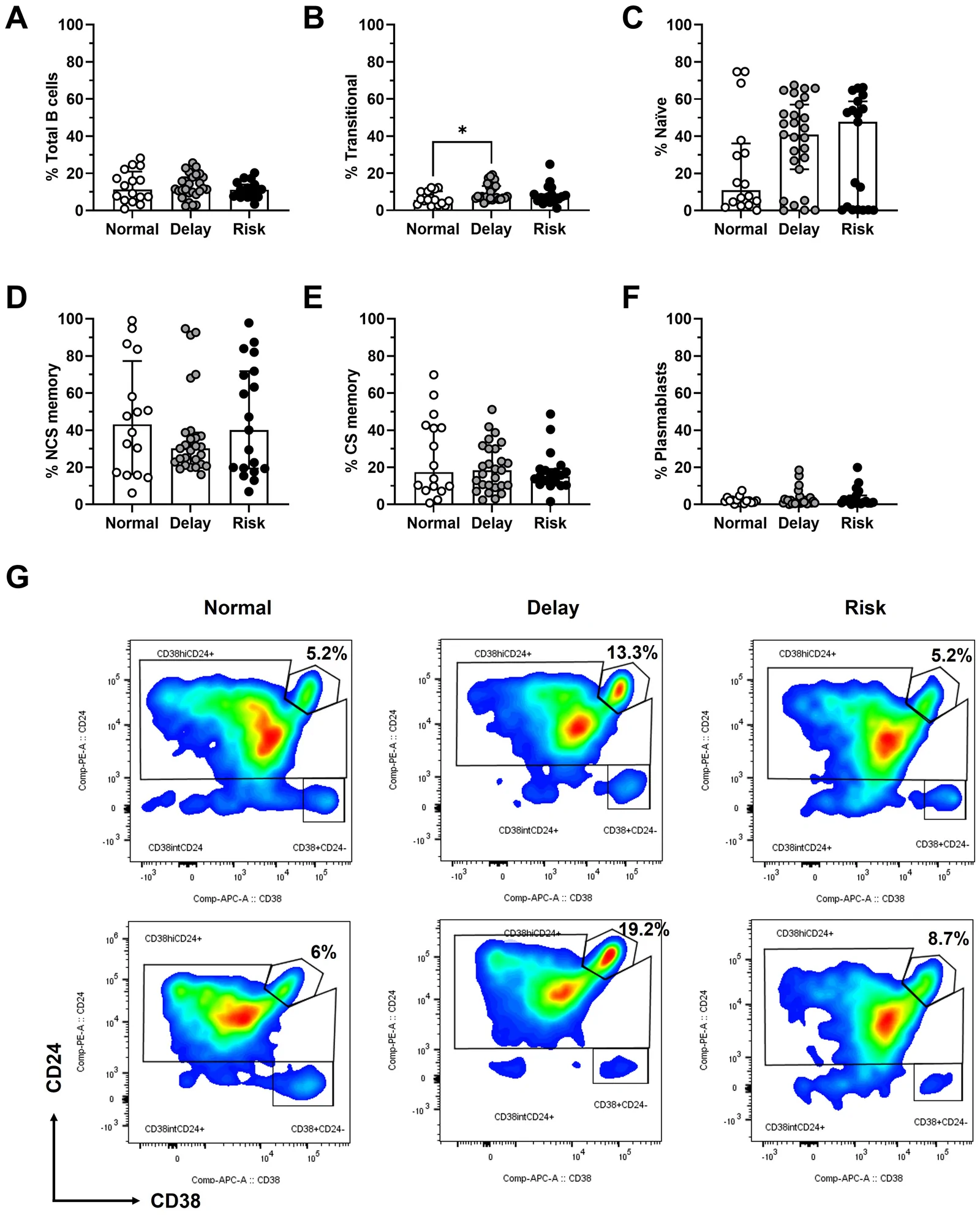
Frequency of peripheral blood B cells from preschoolers with normal, delay and at-risk results in the EDI-II test. **(A)** Total B cells, **(B)** Transitional B cells, **(C)** Naïve B cells, **(D)** Non-class-switched memory B cells (NCS memory), **(E)** Class-switched memory B cells (CS memory), **(F)** Plasmablasts, **(G)** Representative flow cytometry plots of Transitional B cells (CD38^hi^CD24^+^) on total B cells (CD19^+^HLA-DR^+^) from the three study groups, cell frequencies are indicated in the right corner. Data are presented as median with interquartile range, Kruskal-Wallis with Dunn’s multiple comparisons test, *p < 0.05. Normal (*n* = 16); Delay (*n* = 27); Risk (*n* = 19). The full sequential gating strategy for B cell analysis is detailed in **Supplementary Figure 6**.

### 3.6 High-dimensional analysis identifies immune clusters predictive of developmental risk

To depict the immune landscape associated with the EDI-II results (Normal, Delay, and Risk), we performed high-dimensional flow cytometry on peripheral blood mononuclear cells. We applied an automated computational pipeline comprising dimensionality reduction (UMAP), unsupervised metaclustering (FlowSOM/ConsensusClusterPlus), and marker enrichment modeling to identify distinct cell populations within the T-cell, B-cell, and NK-cell compartments.

While the T-cell and NK-cell compartments showed a high degree of overlap between clinical groups (**Supplementary Figure 3**), the B-cell compartment revealed distinct predictive features associated with clinical outcomes (**Supplementary Figure 4**). To identify specific populations predictive of clinical risk, we applied the RAPID algorithm (LASSO-regularized regression) to train a robust predictive model (**Supplementary Figure 9**). The relative frequencies (%) of the 15 B-cell metaclusters (**Supplementary Figure 4A**) were used as input features to predict a binary clinical status (Normal vs. a combined Delay/Risk group) (**Figure 4**).

**Figure 4:**
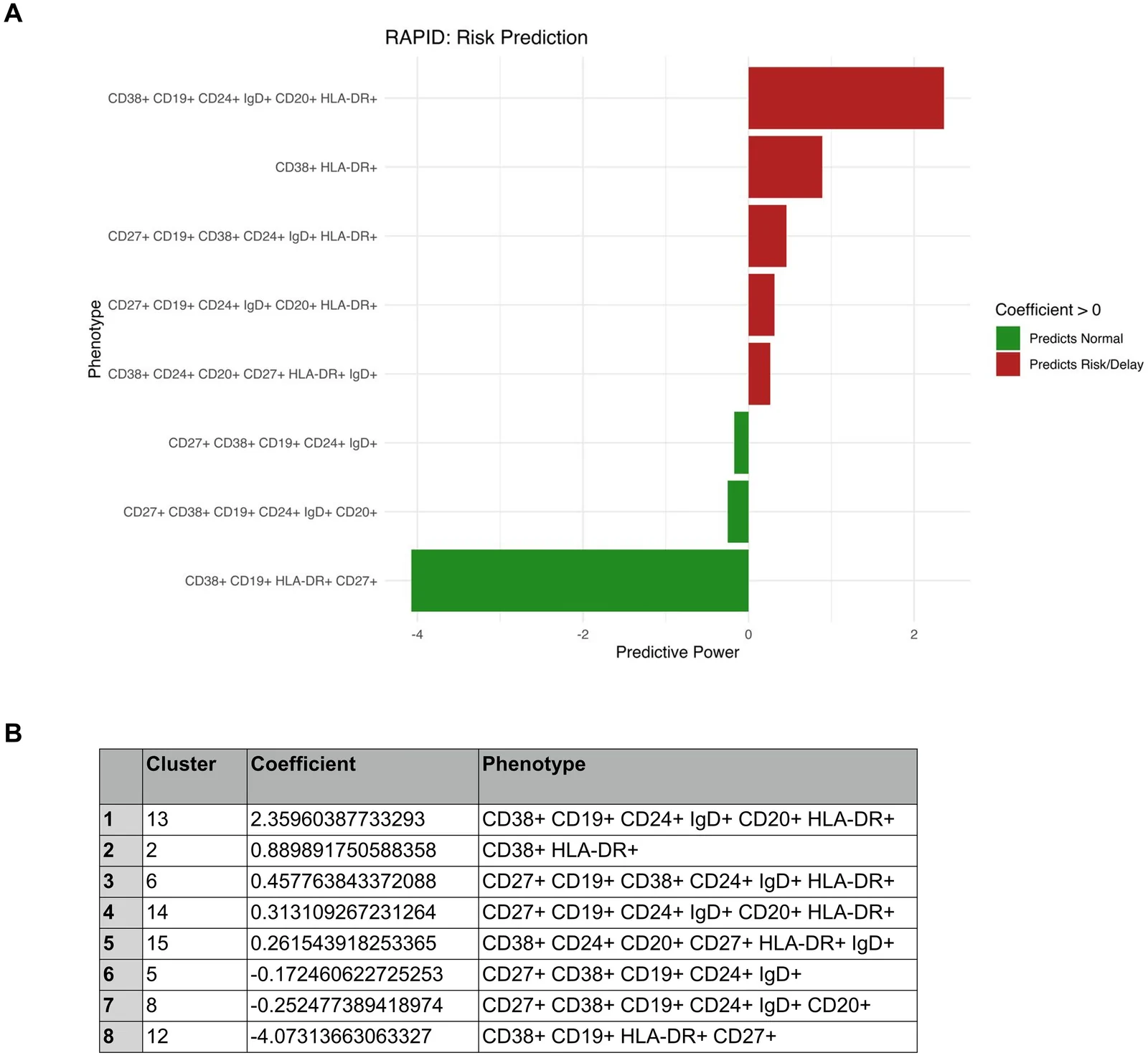
Transitional B cells predict neurodevelopment delay/at-risk in preschoolers born preterm. **(A)** RAPID (LASSO) regression results showing B cell clusters that significantly predict neurodevelopment delay/at-risk in EDI-II test. Bars represent the LASSO regression coefficients for each cell population retained in the final model. The coefficient’s magnitude indicates the “predictive power,” or the strength of its association with the outcome. Red Bars (Positive Coefficients) indicate that a higher frequency of population is associated with an increased log-odds of being in the Risk/Delay group. Green Bars (Negative Coefficients) indicate that a higher frequency of the population is associated with an increased log-odds of being in the Normal group. The final model was selected by minimizing binomial deviance during cross-validation and achieved accuracy of 82.3%, a sensitivity of 97.8%, and a specificity of 37.5%, **(B)** The LASSO regression coefficient’s magnitude indicates the predictive power associated with the outcome. Positive coefficients indicate that a higher frequency of the population is associated with an increased log-odds of being in the Risk/Delay group. Negative coefficients indicate that a higher frequency of the population is associated with an increased log-odd of being in the Normal group. Cluster 13 (CD38^+^CD19^+^CD24^+^IgD^+^CD20^+^HLA-DR^+^, Transitional B cells, coefficient 2.36) strongly predicts Risk/Delay of neurodevelopment, while cluster 12 (CD38^+^CD19^+^HLA-DR^+^CD27^+^, Memory B cells, coefficient -4.07) better predicts Normal neurodevelopment.

The model identified a distinct B-cell signature that stratified the “Normal” status from the “Risk/Delay” status (**Figure 4**). The strongest predictor of the Risk/Delay outcome was Cluster 13 (Coefficient ~2.36, **Figure 4B**; indicated by a red bar in **Figure 4A**), defined as CD38^+^CD19^+^CD24^+^IgD^+^CD20^+^HLA-DR^+^. This phenotype corresponds to transitional B cells, which are immature emigrants from the bone marrow.

Conversely, the model identified Cluster 12 as the strongest predictor of the Normal outcome (Coefficient ~ -4.07, **Figure 4B**; indicated by a green bar in **Figure 4A**). This population displayed a CD38^+^CD19^+^HLA-DR^+^CD27^+^ phenotype, notably lacking IgD and CD24, indicating a Class-Switched Memory B cell profile. Additionally, Cluster 8 (CD27^+^CD38^+^ CD19^+^CD24^+^IgD^+^CD20^+^) and Cluster 5 (CD27^+^CD38^+^CD19^+^CD24^+^IgD^+^) were associated with the Normal outcome.

The LASSO regression coefficient’s magnitude indicates the predictive power associated with the outcome. Positive coefficients indicate that a higher frequency of the population is associated with an increased log-odds of being in the Risk/Delay group, while negative coefficients indicate that a higher frequency of the population is associated with an increased log-odds of being in the Normal group (**Figure 4B**). These results indicate preschoolers with neurodevelopmental impairments are distinguished by a persistence of immature, transitional B-cell phenotypes (Cluster 13), whereas children with normal neurodevelopment exhibit a more mature B-cell profile enriched in class-switched memory cells (Cluster 12). Running this evaluation on our cohort’s training data yielded an Accuracy of 82.26%, a Sensitivity of 97.83%, and a Specificity of 37.50%.

### 3.7 Reduced IP-10 expression specifically characterizes the at-risk phenotype

The analysis of serum concentrations of cytokines, chemokines, and neuroinflammatory molecules revealed a decrease in IP-10 concentrations in the Risk group compared to the Normal neurodevelopment group, and no other significant differences were observed (**Table 4**). IL-1β, IL-2 and IFN-γ were undetectable in preschool children’s samples.

**Table 4:** Serum cytokines, chemokines and neuroinflammatory molecules from studied preschoolers.

Analyte (pg/mL)	Normal (*n* = 16)	Delay (*n* = 24)	Risk (n = 20)	*p*-value
IL-1β	ND	ND	ND	
IL-2	ND	ND	ND	
IL-4	18.1 [11.3-31.9]	17.3 [11.1-24.7]	18.9 [10.9-29.1]	0.908
IL-8	2.3 [0.4-3.3]	2.7 [1-18]	4.14 [1.1-5.3]	0.449
IL-10	3.3 [0.8-4.7]	1.6 [0.6-3.8]	1.3 [0.5-3.2]	0.488
IL-12p70	4.9 [2.7-8.9]	4.3 [2.8-7.3]	4.4 [2.9-9.3]	0.689
IL-17A	6.9 [5.2-10.2]	7.7 [6.1-11]	7.6 [4.9-11]	0.725
IFN-γ	ND	ND	ND	
IP-10	80.7 [60.7-103.5]	68.7 [53.8-109.7]	62.8 [44.6-70.4]*	**0.037**

Analyte	Normal (*n* = 16)	Delay (*n* = 23)	Risk (*n* = 18)	*p*-value
BDNF	11383 [9177-12345]	10881 [9126-11929]	10622 [8546-12115]	0.795
CCL2	121.8 [88.8-167.8]	113.1 [81.4-154.7]	99.7 [82-147.4]	0.523
CX3CL1	3266 [2343-5100]	2678 [2167-3785]	3256 [2917-4886]	0.188
IL-6	65.1 [55.7-73.8]	83.5 [62.2-155.9]	67.5 [51.1-105.2]	0.294
IL-18	248.9 [158.4-379.8]	239.5 [195.7-349]	219.3 [166.3-301.3]	0.864
sRAGE	1261 [836.6-1766]	1121 [852.2-2217]	1350 [1117-1779]	0.587
sTREM-1	503.5 [369.9-948.4]	413.8 [283.4-623.8]	581.4 [350.4-843.9]	0.268
sTREM-2	3467 ± 1174	3372 ± 1265	4182 ± 1349	0.109
TNF-α	29.1 [21.6-55]	41.2 [26.1-71.7]	43.4 [27.9-64.7]	0.536
TGF-β1	78.6 [53.5-122.7]	64.6 [51.3-104.2]	92.5 [61.2-110.9]	0.232
VILIP-1	730.1 [656.3-883.2]	733.2 [584.3-935.1]	852.1 [769.3-974.8]	0.221
VEGF	143.7 [117.4-225.3]	116.9 [99.7-161.8]	117.7 [98.1-165]	0.143
β-NGF	5.2 [4.4-6.2]	5.2 [3.6-7.3]	5.9 [4.5-8.4]	0.511

ND, Not detected. Data are presented as mean ± standard deviation or median [interquartile range]. One-way ANOVA or Kruskal-Wallis tests were used for multiple comparisons. Statistically significant *p*-values (*p* < 0.05) are shown in bold.

### 3.8 Integrative analysis reveals associations between immune signatures, body composition, and neurodevelopment

A correlation analysis was performed between neurodevelopmental domain scores evaluated with the MSCA test (verbal, perceptual-performance, quantitative, general cognitive index, memory and motor), and the anthropometric, body composition and immunological variables. At 48 months, the verbal domain showed a positive correlation with anthropometric indicators (ΣSF, % fat mass, head and abdomen circumferences, subscapular and abdominal skinfolds thickness) and immunological parameters (transitional and NCS memory B cells, IL-10, and VEGF). The perceptual-performance domain was mainly related to adiposity indicators (ΣSF, % fat mass, subscapular skinfold thickness). The quantitative domain showed positive correlations with subscapular skinfold thickness, plasmablasts and NCS memory B cells and a negative correlation with naïve B cells. In the general cognitive index domain positive correlations were observed with markers of adiposity (ΣSF, %vfat mass, abdomen circumference, subscapular and abdominal skinfolds thickness), plasmablasts, NCS memory B cells, and VEGF. The memory domain positively correlated with subscapular skinfold, NCS memory B cells, IL-18 and VEGF. The motor domain showed positive correlations with adiposity indicators (ΣSF, % fat mass, subscapular and abdominal skinfolds thickness) (**Figure 5**, **Supplementary Figure 10**).

**Figure 5:**
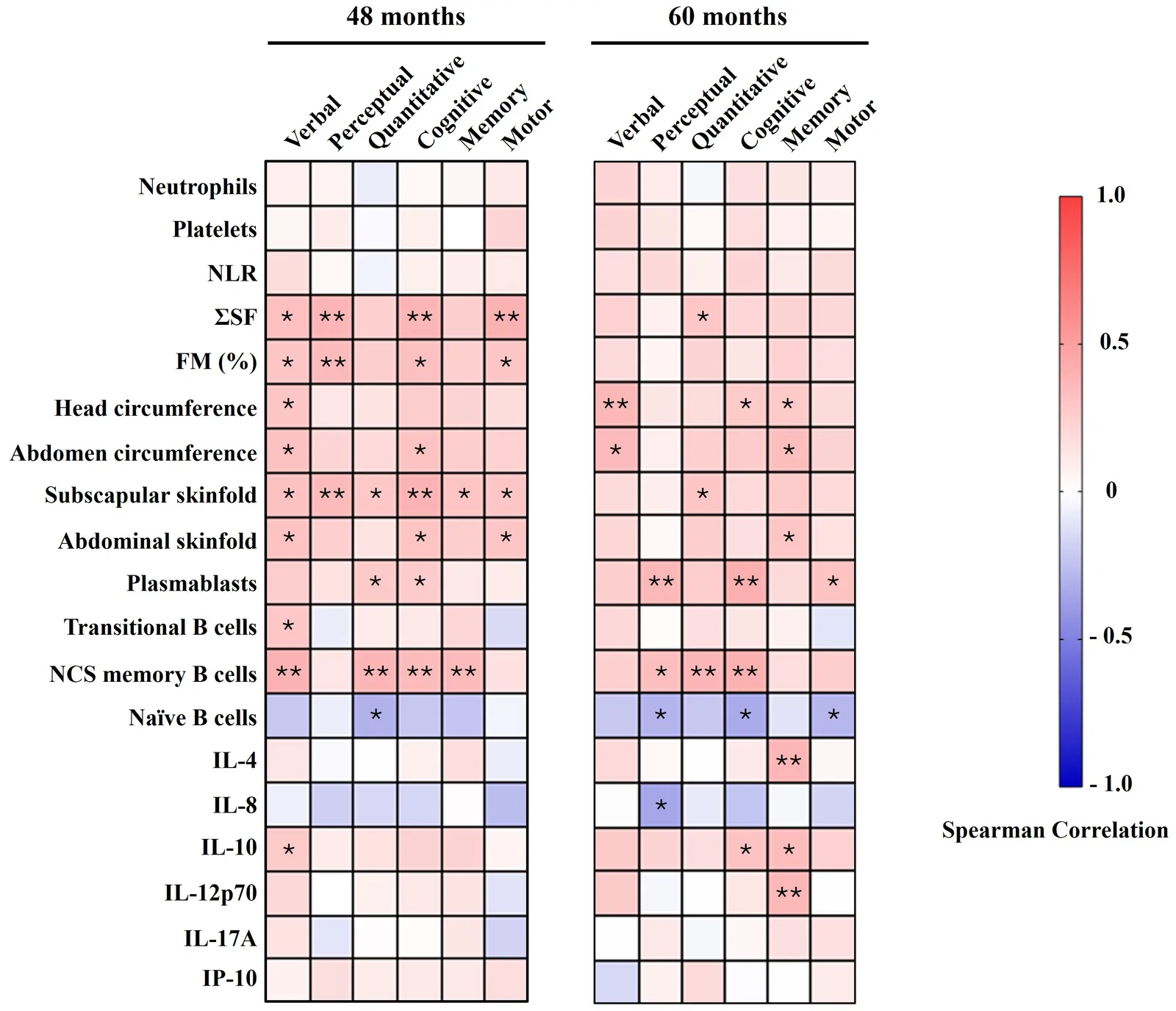
Correlation analysis of MSCA domains with anthropometric and immunological variables. Preschoolers were evaluated with the McCarthy Scales of Children’s Abilities (MSCA) at ages 42-48 (n = 61) and 60-months old (n = 55), and the scalar results obtained in the Verbal, Perceptual-Performance (Perceptual), Quantitative, General Cognitive Index (Cognitive), Memory and Motor domains were analyzed for correlation with Neutrophils and Platelets cell counts, Neutrophil to Lymphocyte Ratio (NLR), Sum of skinfolds thickness (ΣSF), % Fat Mass (FM), Head and Abdomen Circumferences, Subscapular and Abdominal Skinfolds thickness, B cell subsets [Plasmablasts, Transitional B cells, Non class-switched memory B cells (NCS memory B cells) and Naïve B cells], and serum IL-4, IL-8, IL-10, IL-12p70, IL-17A and IP-10. Spearman correlation, positive and negative correlations are shown in red and blue, respectively, *p < 0.05, **p < 0.01.

At 60 months, the verbal domain was associated positively with head and abdomen circumferences, whereas the perceptual-performance domain showed a positive correlation with plasmablasts and NCS memory B cells and a negative correlation with naive B cells and IL-8. The quantitative domain was related to adiposity indicators (ΣSF and subscapular skinfold thickness) and NCS memory B cells. In the general cognitive index domain, positive correlations included head circumference, plasmablasts, NCS memory B cells, and IL-10, and a negative correlation with naive B cells was observed. In the memory domain, positive associations were identified with head and abdomen circumferences, abdominal skinfold thickness, IL-4, IL-10, IL-12p70, and VEGF. The motor domain showed a positive correlation with plasmablasts, and a negative correlation with naïve B cells (**Figure 5**, **Supplementary Figure 10**).

## 4 Discussion

Premature birth exerts profound and potentially lasting effects on child development, influencing body composition, innate and adaptive immune responses, and critical neurological processes such as neurogenesis, myelination, and synaptogenesis (13). Beyond acute neonatal injuries, prematurity is associated with subtle, persistent alterations in white matter microstructure and functional connectivity, which often manifest as motor, cognitive and language impairments later in life (40, 41). Hence, identifying growth patterns and biological markers associated with these outcomes during the preschool years is crucial. Early identification facilitates timely intervention, potentially mitigating long-term neurodevelopmental limitations.

Consistent with literature indicating that preterm children, even those without overt neurological deficits, score lower on cognitive, attention, and executive function tests (21–23), our cohort exhibited developmental vulnerabilities. Using the EDI-II screening tool, we identified delays in gross motor skills, cognition, and language as the most prevalent impairments in preterm preschoolers at 42–48 months old. These results were corroborated by the diagnostic test MSCA, which identified motor, quantitative, verbal and memory as the most vulnerable domains in preschoolers at 48 and 60 months. Our findings align with reports from preterm infant cohorts evaluated with the Bayley-III scales and the INTERGROWTH-21st Neurodevelopment Assessment at 2-years old (19–21, 42), in which motor, cognitive and language impairments were the most frequent outcomes. The persistence of these deficits in our preterm cohort at the preschool period highlights that natural compensatory mechanisms can fail to overcome developmental gaps, as previously shown in other children’s cohorts (43, 44). Our findings with the EDI-II test, highlight the usefulness of this test to quickly and efficiently detect (15 minutes assessment) neurodevelopmental impairments, which is especially important in field work to assess the neurodevelopment of vulnerable child populations (29, 30).

Our initial screening of the preschooler´s immune system status revealed a decrease in neutrophil counts and the Neutrophil-to-Lymphocyte Ratio (NLR) in the Risk group. This parallels findings in cord blood (45) and 2-year-old preterm infants (46), where reduced neutrophils were associated with lower levels of Insulin-like Growth Factor 1 (IGF-1). Moreover, we found reduced platelet counts in children with neurodevelopmental Delay/Risk, and these cells are emerging as critical players in neurodevelopmental disorders such as autism spectrum disorder (ASD) (47). Platelets share common characteristics in subcellular organization and protein composition with neurons (48, 49), thus the link between myeloid lineage suppression and altered neurodevelopment in children warrants further investigation.

Childhood environmental determinants such as nutrition, cognitive stimulation, and indicators of family well-being like maternal educational and socioeconomic status (SES) can act as neuromodulators that shape brain and behavioral development (50–54). In terms of SES and maternal education, our cohort was very homogeneous, most children belonged to low SES families and a maternal schooling median of 12 years. Low SES and maternal education levels have been linked to reduced brain surface area in regions governing language, reading, executive functions and spatial abilities (55). Therefore, while the biological perturbation caused by preterm birth is central, the lack of cognitively stimulating environments, often associated with poverty, may exacerbate these deficits (56, 57).

We previously reported that preterm infants from our cohort had similar feeding practices during hospitalization and all preterm infants were fed human milk (mothers’ own milk or pasteurized donor human milk) either exclusively or in combination with fortified human milk or preterm formula (58). Concerning post-discharge feeding practices, about 80% of preterm infants started solid foods at 6-months corrected age (CA) vs 20% at 6-months chronological age; and 50% of children were fed human milk either exclusively or in combination with formula and 50% were fed exclusively formula at 6-months CA (Unpublished).

At preschool age, the food consumption frequency assessment did not reveal differences between study groups. However, the anthropometric and body composition assessment provided further insight into the systemic nature of the observed developmental impairments. While growth, dietary intake and general metabolic markers appeared similar, we observed a distinct body composition phenotype in children with neurodevelopmental delay and risk. These children exhibited a “leaner phenotype” characterized by reduced abdominal, triceps, and subscapular skinfolds thickness, and lower FM percentage compared with their normal counterparts.

Altered adipose tissue in preterm infants at term-equivalent age, preschool and school age remains controversial (24, 27, 59–62), our findings align with reports observing no excess adiposity in later childhood. Children with neurodevelopmental impairments frequently exhibit reduced FFM and disproportionate fat mass accumulation. Binder et al. reported that higher FFM Z-scores were significantly and independently positively associated with cognitive, language and motor scores in 3-year-old preschool children born extremely preterm (<28 weeks GA) using Bayley-III scales (63). In our cohort, preschool children were born very preterm to moderately preterm (28–34 weeks GA), and we found reduced FM but not in FFM in children with neurodevelopmental impairments. The leaner phenotype observed in children with neurodevelopmental impairments suggests that energy is prioritized toward vital metabolic functions and basic maintenance over adipose storage, or alternatively, reflects distinct adipose compartmentalization programming (64).

Taking together our results suggest that the combination of the EDI-II test, a routine and low-cost lab test such as hematic biometry, and the subscapular skinfold thickness measure hold promise as useful tools for early identification of preterm children at risk of neurodevelopmental impairments.

Regarding immunological status, our study offers novel insights into the “neuro-immune axis” in preterm survivors. While T-cell and NK-cell compartments appeared homeostatically recovered, the B-cell compartment revealed significant alterations that tracked with neurodevelopmental outcomes. Our high-dimensional computational analysis (RAPID/UMAP) identified Cluster 13, a Transitional B-cell phenotype (CD38^+^CD24^+^IgD^+^), as the strongest predictor of the Delay/Risk outcome. Transitional B cells represent an intermediate stage between immature bone marrow B cells and mature naïve B cells; in healthy development, their frequency typically decreases with age (65). The predictive dominance of this immature population in the Delay/Risk group suggests a state of immunological immaturity that parallels the observed neurodevelopmental delays.

The dominance of this immature Transitional B-cell population (CD38^+^CD24^+^IgD^+^) in the Risk/Delay group raises mechanistical questions regarding the neuro-immune axis. Human transitional B (TrB) cells were first described and characterized by a CD24^hi^CD38^hi^ phenotype (66). Subsequently, based on differential CD21 expression, two subsets of human TrB cells with distinct functions have been described (67). In mice, TrB cells can be further subdivided into three subsets representing different maturation statuses (68). In humans, the CD24^hi^CD38^hi^ transitional compartment is highly enriched for regulatory B cells that maintain immune tolerance primarily via IL-10 secretion (69). These studies demonstrate that TrB cells are a heterogeneous population at various developmental stages with diverse functional properties. Because optimal neurodevelopment relies on peripheral immune signals, we hypothesize that this expanded transition pool in delayed children may be functionally stalled, failing to mature and provide adequate neuroprotective cytokine support (such as IL-10) to the developing brain.

Conversely, Cluster 12, characterized as Class-Switched Memory B cells (CD27^+^IgD^-^), was strongly predictive of Normal development. The presence of these cells reflects a “trained” adaptive immune system that has successfully developed immunological memory, a trajectory appropriate for preschool-aged children. Collectively, these findings suggest that preterm preschoolers with neurodevelopmental impairments exhibit systemic maturational lag. They have not only failed to fully develop motor and cognitive domains but also retain an immature immune signature characterized by high transitional B cells and a relative paucity of the memory B cells seen in normally developed children.

Ultimately, this striking dichotomy between the arrested B-cell signature (cluster 13) in delayed children and the mature, class-switched memory profile (cluster 12) in normally developing peers underscores that preterm neurodevelopmental deficits are linked to a systemic lag in the parallel maturation of the adaptive immune system and the central nervous system.

Cytokine, chemokine and neuroinflammatory molecules assessment revealed reduced levels of IP-10 (CXCL10) in the Risk group. IP-10 is a potent proinflammatory chemokine whose gene expression is primarily and strongly driven by IFN-γ, a cytokine that was undetectable in our cohort together with IL-1β and IL-2. Our findings are consistent with a recent study in which 39 neuroinflammation biomarkers were evaluated in school children born preterm with neurodevelopmental impairments and reports IL-1β and IFN-γ below the detection range (12). The alteration in IP-10, a chemokine involved in Th1 trafficking, suggests that while overt inflammation may have been resolved, subtle dysregulation in immune signaling pathways persists. A negative association of IP-10 and social behaviors has been reported in 3.8–6 years old children with ASD (70).

In this study we performed a comprehensive neurodevelopmental assessment using both screening (EDI-II) and diagnostic (MSCA) tools, alongside a detailed high-dimensional immunological characterization and meticulous body composition analysis. Our results suggest that preterm birth exerts long-term effects not only on neurodevelopment but also on specific immune and body composition profiles. The association between reduced subcutaneous adiposity, the persistence of immature B-cell phenotypes, and neurodevelopmental delays highlights the systemic nature of prematurity. Integrating these body composition and immunological biomarkers into pediatric follow-up could facilitate the early identification of children at risk, enabling timely interventions to support their long-term cognitive and physical health.

## 5 Conclusion

Preterm preschoolers with neurodevelopmental impairments showed a systemic profile of biological immaturity (reduced subcutaneous adiposity, lower neutrophil and platelet counts and a persistence of transitional B cells). If confirmed in independent and larger cohorts, body composition and B-cell maturation markers could complement developmental screening for the early identification of children at risk.

## 6 Limitations

The study has several limitations that should be considered for data interpretation. First, the relatively small sample size precluded sex-stratified analyses, and while our cohort size is comparable to previous immunophenotyping and neurodevelopmental assessment studies in preterm infants, these findings require replication in larger cohorts. Second, the classification of neurodevelopmental groups was based on the EDI-II test, a screening instrument validated for Mexican children (29, 30). The validation of EDI-II test was performed with the Bayley Scales of Infant and Toddler Development, Third Edition (Bayley-III), and with The Battelle Developmental Inventory, Second Edition (BDI-2) (71), which have been validated and widely used for neurodevelopmental assessment of children from the United States, Latin America and European countries. Third, data on early-intervention therapies (i.e. physical therapy, language stimulation) received by children between hospital discharge and neurodevelopmental assessment at preschool age, were not systematically collected and analyzed. However, most children were followed up after hospital discharge at the Pediatric Follow-up Department at INPer and were included in Early-Stimulation or Rehabilitation programs according to their neurodevelopmental status. Fourth, due to sample size limitations, the LASSO predictive model’s accuracy, sensitivity, and specificity were calculated on the training set rather than an independent hold-out test set; thus, this specific B-cell immune signature requires future validation in an independent longitudinal cohort. Finally, our initial high-dimensional flow cytometry panel was restricted to phenotypic surface markers. We lacked the expanded phenotypic mapping (e.g., CD21, CD44, CD93, CD23), transcriptional profiling, and functional assays (such as autoreactivity or IL-10 cytokine secretion) needed to fully characterize Cluster 13. Determining the specific cytokine profiles and transcriptional regulation of this distinct preterm B-cell subset are immediate avenues for our future mechanistic research in dedicated, newly recruited cohorts.

## Data Availability

The original contributions presented in the study are included in the article/**Supplementary Material**. Further inquiries can be directed to the corresponding authors. The R scripts used for the FlowSOM clustering and LASSO regularized regression are available on GitHub at https://github.com/omedinac/TrB-signatures.
