# Supplementary material for: Immune and body composition signatures of neurodevelopmental impairments in preterm preschoolers
Source: Front Immunol. 2026 Oct 19;17:1941190. doi: 10.3389/fimmu.2026.1941190 (PMC13724492; doi:10.3389/fimmu.2026.1941190)
Supplement: Supplementary file 1 [file SupplementaryFile1.docx]

**Supplementary Information**

**Supplementary Figure 1**. Frequency of peripheral blood NK cells from preschoolers born preterm with normal, delay and at-risk results in the EDI-II test. (A) CD56^dim^CD16^+^ NK cells, (B) CD56^-^CD16^+^ NK cells, and (C) CD56^hi^CD16^-^ NK cells. Data are presented as median with interquartile range, Kruskal-Wallis with Dunn’s multiple comparisons test. Normal (*n* = 15); Delay (*n* = 27); Risk (*n* = 17).

**Supplementary Figure 2.** Frequency of peripheral blood T cells from preschoolers born preterm with normal, delay and at-risk results in the EDI-II test. (A) Total T cells, (B) CD4^+^ T cells, (C) CD8^+^ T cells, (D-E) CD4^+^ T cells subpopulations: (D) Naïve, (E) Central memory (T_CM_), (F) Effector memory (T_EM_), and (G) Effector memory re-expressing CD45RA (T_EMRA_), (H-K) CD8^+^ T cells subpopulations: (H) Naïve, (I) T_CM_, (J) T_EM_, and (K) T_EMRA_. Data are presented as median with interquartile range, Kruskal-Wallis with Dunn’s multiple comparisons test. Normal (*n* = 16); Delay (*n* = 24); Risk (*n* = 19).

**Supplementary Figure 3.** Characterization of T cells and NK cells compartments. (A) Uniform Manifold Approximation and Projection (UMAP) visualization of peripheral blood T cells colored by EDI-II test results (Normal, Delay, Risk), (B) Principal Component Analysis (PCA) of T cells cluster frequencies, (C) UMAP visualization of peripheral blood NK cells colored by EDI-II test results, (D) PCA of NK cells cluster frequencies.

**Supplementary Figure 4.** Characterization of the B cell compartment. (A) Heatmap showing median marker expression (arcsinh transformed) for 15 peripheral blood B cells metaclusters (left) and their phenotype labels (right), (B) Uniform Manifold Approximation and Projection (UMAP) visualization colored by EDI-II test results (Normal, Delay, Risk), (C) Principal Component Analysis (PCA) of B cells cluster frequencies.

**Supplementary Figure 5.** Peripheral blood T cells flow cytometry gating strategy. Doublet exclusion (FSC-H vs FSC-A were selected and singlets were gated by selection of the diagonal population), debris exclusion (FSC-A vs SSC-A were selected and lymphocytes were gated by selection of FSC-A^low^SSC-A^low^ cells), and dead cells exclusion (FSC-A vs Zombie Aqua were selected and live cells were gated by selection of Zombie Aqua^-^FSC-A^low^ cells) were performed to select single live lymphocytes. Next, total T cells were gated by selection of CD3^+^FSC-A^low^ cells, and CD4^+^ and CD8^+^ T cell subpopulations were gated by selection of CD4^+^CD8^-^ and CD8^+^CD4^-^ cells, respectively. Then, naïve (CD45RA^+^CCR7^+^), T central memory (T_CM_, CD45RA^-^CCR7^+^), T effector memory (T_EM_, CD45RA^-^CCR7^-^), and T effector memory re-expressing CD45RA (T_EMRA_, CD45RA^-^CCR7^-^) subsets within CD4^+^ and CD8^+^ T cell subpopulations were gated based on CD45RA and CCR7 expression.

**Supplementary Figure 6.** Peripheral blood B cells flow cytometry gating strategy. Doublet exclusion (FSC-H vs FSC-A were selected and singlets were gated by selection of the diagonal population), debris exclusion (FSC-A vs SSC-A were selected and lymphocytes were gated by selection of FSC-A^low^SSC-A^low^ cells), and dead cells exclusion (FSC-A vs Zombie Aqua were selected and live cells were gated by selection of Zombie Aqua^-^FSC-A^low^ cells) were performed to select single live lymphocytes. Next, total B cells were gated by selection of CD19^+^HLA-DR^+^ cells, and transitional B cells (CD38^hi^CD24^+^) and plasmablasts (CD38^+^CD24^-^) were gated based on CD38 and CD24 expression. Then, CD38^dim^CD24^+^ cells were selected and naïve B cells (IgD^+^CD27^-^), non-class-switched memory B cells (NCS memory, IgD^+^CD27^+^), and class-switched memory B cells (CS memory, IgD^-^CD27^+^) were gated based on IgD and CD27 expression.

**Supplementary Figure 7**. Peripheral blood NK cells flow cytometry gating strategy. Doublet exclusion (FSC-H vs FSC-A were selected and singlets were gated by selection of the diagonal population), debris exclusion (FSC-A vs SSC-A were selected and lymphocytes were gated by selection of FSC-A^low^SSC-A^low^ cells), and dead cells exclusion (FSC-A vs Zombie Aqua were selected and live cells were gated by selection of Zombie Aqua^-^FSC-A^low^ cells) were performed to select single live lymphocytes. Then, CD3-CD14-CD19- cells were selected, and CD56^hi^CD16^-^, CD56^dim^CD16^+^ and CD56^-^CD16^-^ NK cells were gated based on CD56 and CD16 expression.

**Supplementary Figure 8.** McCarthy Scales of Children´s Abilities (MSCA) score system. General Cognitive Index (GCI), Standard Deviation (SD).

**Supplementary Figure 9**. Binomial Deviance. The plot shows how the model error (deviance) changes as the regularization strength (λ) increases. X-axis (Log Lambda) shows the strength of the penalty. To the right (high lambda), more coefficients are forced to zero. To the left (low lambda), more are included. Y-axis (Binomial Deviance) shows the average model error across the cross-validation folds. Lower is better. Left dotted line (lambda.min) represents the lambda value that gives the absolute minimum error. Right dotted line (lambda.1se) represents a slightly more regularized (simpler) model that is within one standard error of the minimum.

**Supplementary Figure 10.** Correlation analysis of MSCA domains with immunological variables. Preschoolers were evaluated with the McCarthy Scales of Children's Abilities (MSCA) at ages 42-48 (n = 61) and 60 months old (n = 55), and the scalar results obtained in the Verbal, Perceptual-Performance (Perceptual), Quantitative, General Cognitive Index (Cognitive), Memory and Motor domains were analyzed for correlation with serum IL-6, TGF-β1, BDNF, CCL2 (MCP-1), CX3CL1, IL-18, sRAGE, sTREM-1, sTREM-2, TNF-α, VEGF, VILIP-1, and β-NGF. Spearman correlation, positive and negative correlations are shown in red and blue, respectively, **p* < 0.05, ***p* < 0.01.

| **Supplementary Table 1**  Results from EDI-II test neurodevelopmental domains at 42-48 months old (*n* = 65) | | | |
| --- | --- | --- | --- |
| Domain | **Normal** | **Delay** | **Risk** |
| Cognitive (%) | 43 (66.1) | 20 (30.8) | 2 (3.1) |
| Language (%) | 43 (66.1) | 9 (13.8) | 13 (20.0) |
| Gross motor (%) | 35 (53.8) | 29 (44.6) | 1 (1.5) |
| Fine motor (%) | 48 (73.8) | 14 (21.5) | 3 (4.6) |
| Social (%) | 51 (78.5) | 14 (21.5) | 0 |

| **Supplementary Table 2**  Results from MSCA test neurodevelopmental domains at 42-48 months old (*n* = 61) | | | | |  |
| --- | --- | --- | --- | --- | --- |
| Domain | **Normal** | **Delay** | **Risk** | **High/Advanced** | |
| Verbal (%) | 32 (52.4) | 18 (29.5) | 6 (9.8) | 5 (8.2) |  |
| Perceptual-Performance (%) | 38 (62.3) | 12 (19.7) | 2 (3.3) | 9 (14.7) |  |
| Quantitative (%) | 32 (52.4) | 17 (27.9) | 12 (19.7) | 0 |  |
| General Cognitive Index (%) | 35 (57.4) | 9 (14.7) | 10 (16.4) | 7 (11.5) |  |
| Memory (%) | 44 (72.1) | 12 (19.7) | 5 (8.2) | 0 |  |
| Motor (%) | 26 (42.6) | 27 (44.3) | 5 (8.2) | 3 (4.9) |  |

| **Supplementary Table 3**  Results from MSCA test neurodevelopmental domains at 60 months old (n = 55) | | | | |
| --- | --- | --- | --- | --- |
| Domain | **Normal** | **Delay** | **Risk** | **High/Advanced** |
| Verbal (%) | 37 (67.3) | 11 (20) | 1 (1.8) | 6 (10.9) |
| Perceptual-Performance (%) | 40 (72.7) | 10 (18.2) | 1 (1.8) | 4 (7.3) |
| Quantitative (%) | 33 (60) | 16 (29.1) | 6 (10.9) | 0 |
| General Cognitive Index (%) | 40 (72.7) | 13 (23.6) | 2 (3.6) | 0 |
| Memory (%) | 37 (67.3) | 18 (32.7) | 0 | 0 |
| Motor (%) | 30 (54.5) | 21 (38.2) | 4 (7.3) | 0 |

| **Supplementary Table 4**  Demographic, clinical, and maternal data from studied preschoolers | | | | |
| --- | --- | --- | --- | --- |
| **Neonatal Variable** | **Normal (*n* = 17)** | **Delay (*n* = 27)** | **Risk (*n* = 21)** | ***p*-value** |
| Programmed C-section (%) | 2 (11.8) | 4 (14.8) | 7 (33.3) | 0.205 |
| Emergency C-section (%) | 15 (88.2) | 16 (59.2) | 13 (61.9) | 0.106 |
| Eutocic birth (%) | 0 | 4 (14.8) | 1 (4.8) | 0.207 |
| Dystocic birth (%) | 0 | 3 (11.1) | 0 | 0.178 |
| Singleton (%) | 10 (58.8) | 19 (70.4) | 13 (61.9) | 0.702 |
| Twins (%) | 6 (35.3) | 6 (22.2) | 5 (23.8) | 0.603 |
| Triplets (%) | 1 (5.9) | 2 (7.4) | 3 (14.3) | 0.651 |
| Apgar 1 min | 8 [6.5-8] | 7 [5-8] | 7 [4.5-8] | 0.240 |
| Apgar 5 min | 9 [8-9] | 9 [8-9] | 9 [8-9] | 0.509 |
| **Maternal Variable** | **Normal (*n* = 17)** | **Delay (*n* = 27)** | **Risk (*n* = 21)** | ***p*-value** |
| Low SES (%) | 17 (100) | 19 (70.4) | 20 (95.2) | **0.009** |
| Medium SES (%) | 0 | 8 (29.6) | 1 (4.8) | **0.009** |
| Years of education | 12 [9.75-16] | 12 [9-16] | 12 [9-12] | 0.364 |
| Single (%) | 2 (11.8) | 5 (18.5) | 8 (38.1) | 0.166 |
| Married (%) | 8 (47) | 9 (33.3) | 6 (28.6) | 0.475 |
| Consensual union (%) | 7 (41.2) | 13 (48.1) | 7 (33.3) | 0.586 |
| SES (socioeconomic status). Data are presented as mean ± standard deviation or median [interquartile range]. One-way ANOVA or Kruskal-Wallis tests were used for continuous variables. Fisher's exact test or Chi-Square test were used for categorical variables. Statistically significant *p*-values (*p* < 0.05) are shown in bold. | | | | |

| **Supplementary Table 5**  Food consumption frequency assessment from studied preschoolers | | | | |
| --- | --- | --- | --- | --- |
| **Food group**  **(Days/Week)** | **Normal**  **(*n* = 17)** | **Delay**  **(*n* = 27)** | **Risk**  **(*n* = 21)** | ***p*-value** |
| Vegetables | 4.5 [3-5.5] | 5 [3-7] | 4 [4-7] | 0.722 |
| Fruits | 7 [7-7] | 7 [6-7] | 7 [5.5-7] | 0.296 |
| Animal protein | 5.5 [4-7] | 6 [4-7] | 7 [4.5-7] | 0.538 |
| Legumes | 3.5 [2-7] | 2 [1-4] | 3 [1-3.5] | 0.215 |
| Sugar | 4 [3-7] | 4.5 [2-7] | 7 [4-7] | 0.152 |
| Cereal with fat | 2.5 [1-4.2] | 3 [2-4] | 3 [2-4] | 0.549 |
| Sugary drinks | 3.5 [0.0-5] | 2.5 [1-4.7] | 4 [1.5-7] | 0.449 |
| Tea | 0.0 [0.0-1.2] | 0.0 [0.0-1] | 0.0 [0.0-1] | 0.639 |
| Meals per day | 4 [4-5] | 5 [4-5] | 5 [4-5] | 0.549 |
| Water (mL/day) | 825 [550-1050] | 500 [277.5-937.5] | 750 [480-1000] | 0.389 |
| Data are presented as median [interquartile range]. Kruskal-Wallis test was used for multiple comparisons. | | | | |

| **Supplementary Table 6**  Blood chemistry from studied preschoolers | | | | |
| --- | --- | --- | --- | --- |
| **Parameters**  **(mg/dL)** | **Normal**  **(*n* = 16)** | **Delay**  **(*n* = 25)** | **Risk**  **(*n* = 21)** | ***p*-value** |
| Glucose | 76.7 ± 10.9 | 65.5 ± 17.4 | 70.1 ± 12.2 | 0.058 |
| Triglycerides | 60 [51.7-81.2] | 55 [46-87] | 68 [54.5-82.5] | 0.630 |
| Cholesterol | 149.3 ± 20.2 | 140.2 ± 35.1 | 156.6 ± 32.4 | 0.207 |
| HDL-Cholesterol | 48.5 ± 9.7 | 44.8 ± 9.1 | 47.3 ± 15.5 | 0.593 |
| Creatinine | 0.4 [0.2-0.5] | 0.3 [0.2-0.5] | 0.3 [0.2-0.5] | 0.511 |
| Uric acid | 4.0 [3.1-4.7] | 3.2 [2.7-4.1] | 3.7 [3.2-4] | 0.217 |
| Urea | 26.2 [23.2-28.5] | 26.2 [23.8-32.3] | 27.4 [23-30.5] | 0.716 |
| Data are presented as mean ± standard deviation or median [interquartile range]. One-way ANOVA or Kruskal-Wallis tests were used for multiple comparisons. HDL (High-Density Lipoprotein) | | | | |

**Supplementary Methods**

**Neurodevelopmental Assessment**

The Child Development Evaluation Test second edition (EDI-II) was validated for Mexican children using the Battelle Developmental Inventory Second Edition and demonstrates 81% sensitivity (95% CI: 75-86%) and 61% specificity (95% CI: 54-67%) for detection of developmental deficits. The test was designed to detect developmental lags and risk of developmental delay and involves direct observation of the child's performance and a structured interview to primary caregivers. In the traffic light system, green (Normal Development) indicates that children successfully performed the activities for the corresponding age group without alarm signs; yellow (Developmental Lag, hereafter referred as Delay) indicates that not all activities for the corresponding age group were successfully performed but those for the preceding age did; and red (Risk of developmental delay, hereafter referred as Risk) indicates that neither the activities of the preceding and corresponding age group were fully performed or that the child presents neurological alarm signs. The global result of the EDI-II test reflects child performance in the 5 evaluated domains (gross motor, fine motor, language, social, and cognitive). If a child scores green in the 5 domains, the global result is Normal neurodevelopment, whereas if one or more domains score yellow or red, the overall result is Developmental Lag or Risk of developmental delay, respectively.

**UMAP Analysis**

Computational Flow Cytometry Analysis

Data Preprocessing and Transformation All computational analyses were performed using the R statistical programming environment (v4.5.2). Raw Flow Cytometry Standard (.fcs) files were imported and processed using the flowCore and CATALYST packages. To address batch-specific labeling variations, channel names were algorithmically homogenized to standardized antigen identifiers prior to merging. Fluorescence intensities were normalized using an inverse hyperbolic sine (arcsinh) transformation with a cofactor of 150 to reduce skewness and handle negative values typical of flow cytometry data.

Dimensionality Reduction and Clustering

High-dimensional visualization was performed using Uniform Manifold Approximation and Projection (UMAP) on a downsampled subset of events (1,000 cells per sample) to visualize the global immune landscape. For quantitative analysis, unsupervised clustering was performed using the FlowSOM algorithm integrated within CATALYST. Cells were grouped into a 10×10 grid of self-organizing maps (SOM) and subsequently merged into 15 metaclusters per panel (T, B, and NK cells) using ConsensusClusterPlus metaclustering to capture biologically relevant subpopulations.

Automated Phenotype Annotation

To ensure reproducible and unbiased population identification, metaclusters were annotated using a custom native auto-phenotyping algorithm. Median marker expression (arcsinh-transformed) was calculated for every metacluster. Markers with a median expression threshold ≥2.0 were classified as “positive” (+), while markers below this threshold were classified as negative. Populations were automatically labeled based on their combinatorial expression profile.

Differential Abundance and Trend Analysis

Statistical analysis of population frequencies was performed using the *diffcyt* framework. Differential abundance (DA) of cell populations between clinical groups (Normal vs. Risk/Delay) was assessed using generalized linear models (GLM) via *edgeR*, with Benjamini-Hochberg (FDR) correction for multiple testing (FDR < 0.05). To characterize immune alterations associated with the severity of the clinical condition, a Spearman rank correlation analysis was performed. Clinical status was treated as an ordinal variable (Normal = 1, Delay = 2, Risk = 3). Cell populations showing a significant monotonic trend (p<0.05) across these stages were identified as tracking with clinical severity.

Risk Assessment Population Identification (RAPID)

To identify a minimal immune signature predictive of clinical outcome, we applied the RAPID (Risk Assessment Population IDentification) algorithm using LASSO (Least Absolute Shrinkage and Selection Operator) regularized logistic regression via the *glmnet* package. Cluster frequencies were used as input features to predict binary clinical status (Normal vs. Risk/Delay). The model was trained using k-fold cross-validation (with k adapted to sample size) to determine the optimal regularization parameter (𝛌). Clusters with non-zero coefficients at 𝛌 were identified as the predictive immune signature. This approach selects the most robust predictors while penalizing non-informative features to prevent overfitting.
